# Supplementary material for: SMC5/6-Mediated Plasmid Silencing is Directed by SIMC1-SLF2 and Antagonized by LT
Source: bioRxiv. 2025 Mar 28:2025.03.27.645818. Preprint. [Version 1] doi: 10.1101/2025.03.27.645818 (PMC11974782; doi:10.1101/2025.03.27.645818)
Supplement: 1 [file NIHPP2025.03.27.645818v1-supplement-1.pdf]

# Supplementary figures:

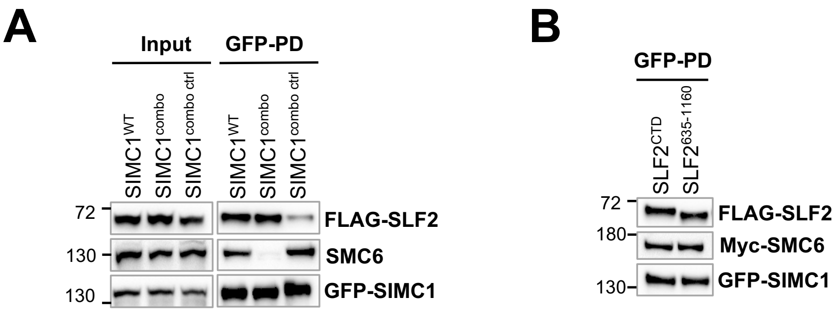


**Figure 1 - figure supplement 1.**

**(A)** Western blot of GFP-trap immunoprecipitation from HEK293 cells transiently transfected respectively with GFP-SIMC1, GFP-SIMC1 combo mutant or GFP-SIMC1 combo control mutant in combination with FLAG-SLF2$^{CTD}$ and Myc-SMC6. Signals were detected using FLAG, SMC6 and GFP antibodies. **(B)** Western blot of GFP-trap immunoprecipitation from HEK293 cells transiently transfected with either FLAG-SLF2$^{CTD}$ or FLAG-SLF2$^{635-1160}$ co-transfected with GFP-SIMC1 and Myc-SMC6. Signals were visualized using FLAG, Myc and GFP antibodies.

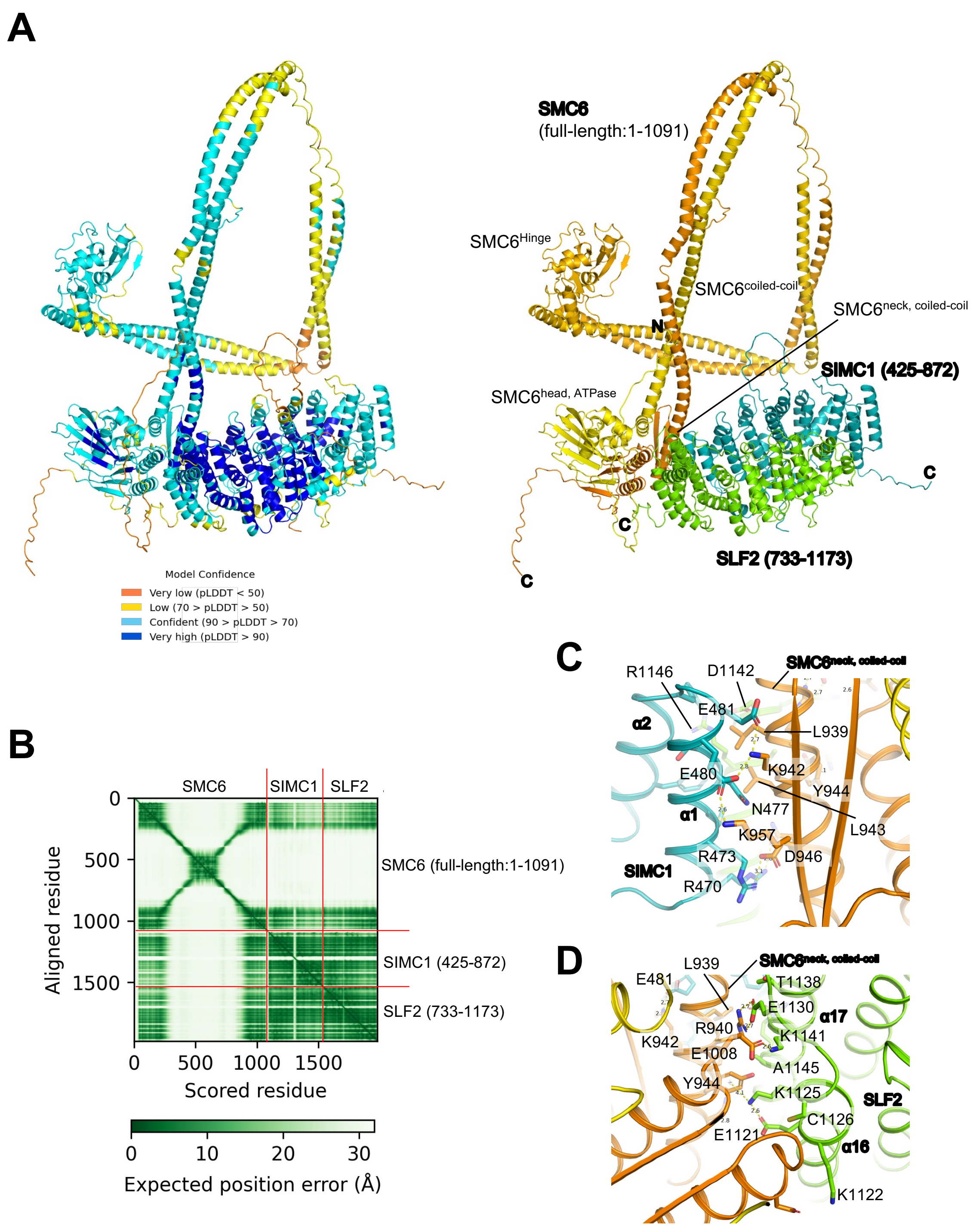


**Figure 2 - figure supplement 1**

**(A)** The AlphaFold-Multimer model of the SIMC1$^{Nse5}$ (425-872)-SLF2$^{Nse6}$ (733-1173)-SMC6 (1-1091) complex. The entire model is shown, colored according to pLDDT values (left) or in the same manner as in **Figure 2A**. **(B)** The Predicted Aligned Error (PAE) plot of the AlphaFold-Multimer prediction. **(C, D)** Close-up views of the SMC6 neck-SIMC1 (C) and SMC6 neck-SLF2 (D) interfaces.

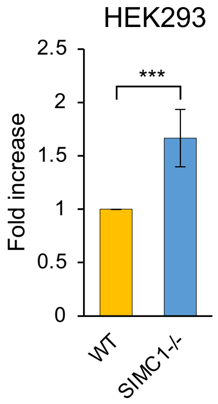


**Figure 3 - figure supplement 1**
HEK293 WT or SIMC1$^{-/-}$ cells were transiently transfected with GFP reporter and GFP intensity was assessed 72 hours later by FACS. Data are the average ± s.d. from n=6 independent experiments, two-tailed unpaired t-test; (***) p<0.0005).

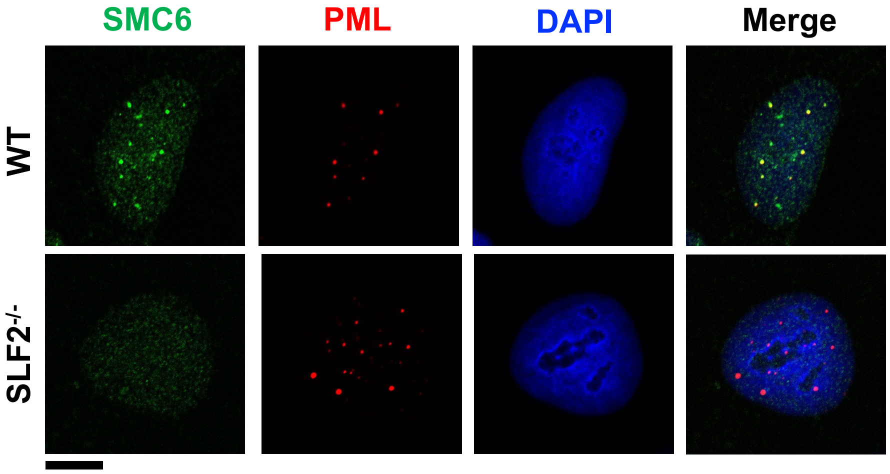


**Figure 5 - figure supplement 1**
Representative immunofluorescence images of U2OS WT or SLF2$^{-/-}$ cells fixed and stained with SMC6 (green) and PML (red) antibodies along with DAPI (blue). Scale bar 10 μm.

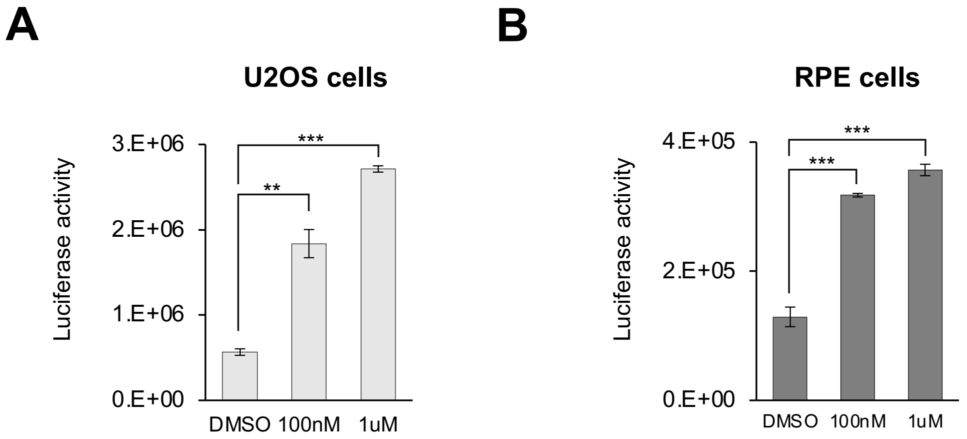


**Figure 5 - figure supplement 2**
Arbitrary luminescence units of U2OS (**A**) or RPE (**B**) cells expressing transfected pLuc reporter. Cells were treated with TAK-981 or DMSO at the time of transfection. Means and error bars (s.d.) were derived from a minimum of n=3 independent transfections representing biological replicates. Two-tailed unpaired t-test was performed (($**$) $p<0.005$; ($***$) $p<0.0005$).

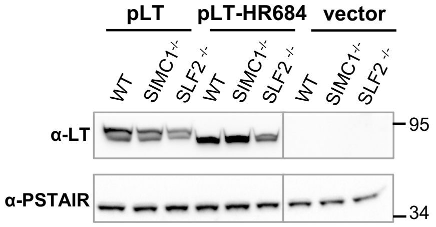


**Figure 5 - figure supplement 3**

Immunoblot from U2OS WT, SIMC1$^{-/-}$ or SLF2$^{-/-}$ cells stably expressing large T antigen (LT), LT-HR684 variant or have an empty vector integrated, respectively. PSTAIR serves as a loading control.

**List of Supplementary files**

Figure 1—figure supplement 1. A, B

Figure 2—figure supplement 1. A-D

Figure 3—figure supplement 1

Figure 5—figure supplement 1.

Figure 5—figure supplement 2. A, B.

Figure 5—figure supplement 3.

**List of source data**

Figure 1 – source data 1. Full and unedited blots corresponding to panel C.

Figure 2 – source data 1. Full and unedited blots corresponding to panel C.

Figure 3 – source data 1. GFP transcripts qPCR corresponding to panel A.

Figure 3 – source data 2. GFP FACS corresponding to panel B.

Figure 4 – source data 1. GFP FACS corresponding to panel B.

Figure 5 – source data 1. GFP FACS corresponding to panel A.

Figure 5 – source data 2. GFP FACS corresponding to panel B.

Figure 5 – source data 3. Full and unedited blots corresponding to panel C.

Figure 5 – source data 4. GFP FACS corresponding to panel D.

Figure 1 – figure supplement 1 – source data 1. Full and unedited blots corresponding to panel A.

Figure 1 – figure supplement 1 – source data 2. Full and unedited blots corresponding to panel B.

Figure 3 – figure supplement 1 - source data 1. GFP FACS.

Figure 5 – figure supplement 2 - source data 1. Luciferase measurement corresponding to panel A.

Figure 5 – figure supplement 2 - source data 2. Luciferase measurement corresponding to panel B.

Figure 5 – figure supplement 3 - source data 1. Full and unedited blots.
